# Randomised controlled trial to test the effectiveness of a locally-produced ready-to-use supplementary food (RUSF) in preventing growth faltering and improving micronutrient status for children under two years in Cambodia: a study protocol

**DOI:** 10.1186/s12937-018-0346-x

**Published:** 2018-03-16

**Authors:** Bindi Borg, Seema Mihrshahi, Mark Griffin, Daream Sok, Chamnan Chhoun, Arnaud Laillou, Jacques Berger, Frank T. Wieringa

**Affiliations:** 1 School of Public Health, Faculty of Medicine, University of Sydney, Sydney, Australia; 2 School of Public Health, University of Queensland, Brisbane, Australia; 3 Department of Nutrition, Exercise and Sports, Faculty of Science, University of Copenhagen, Copenhagen, Denmark; 4 Department of Fisheries Post-Harvest Technologies and Quality Control, Fisheries Administration, Ministry of Agriculture, Forestry and Fisheries, Phnom Penh, Cambodia; 5 Child Survival and Development Section, UNICEF, Phnom Penh, Cambodia; 6 UMR Nutripass, Institut de Recherche pour le Développement, IRD/UM/SupAgro, Montpellier, France; 7 c/o Pascal Marino, European Union Delegation in Cameroon, BP 847 Yaoundé, Cameroon

**Keywords:** Ready-to-use supplementary food (RUSF), Lipid-based nutrient supplement (LNS), Childhood malnutrition, Growth faltering, Corn-Soy Blend Plus Plus (CSB++), Supercereal Plus, Sprinkles micronutrient powders, Cambodia

## Abstract

**Background:**

Existing ready-to-use supplementary and therapeutic foods (RUSFs and RUTFs) have had limited acceptance and effectiveness in Cambodia. This has hampered the treatment and prevention of child malnutrition. An innovative, locally produced, multiple micronutrient fortified lipid-based nutrient supplement (LNS) has been developed for use as an RUSF. Unlike most RUSFs, which contain milk, this product contains fish as the animal protein. Few RUSFs have been formulated using non-milk animal-source foods and they have not been widely tested. An acceptability trial that was conducted on this novel RUSF in June 2015 demonstrated that children will eat the RUSF and that caregivers will feed it to their children. The current trial aims to evaluate the effectiveness of the RUSF in preventing growth faltering and improving micronutrient status in Cambodian children.

**Methods and analysis:**

This trial is a six-month, prospective, cluster randomised, non-blinded controlled trial among infants in peri-urban Phnom Penh. The trial aims to establish the superiority of the novel RUSF, compared to three alternatives (Corn-Soy Blend Plus Plus (CSB++) and Sprinkles micronutrient powders as active comparators, and the unimproved diet as a control). The allocation ratio is 1:1. Healthy children (N = 540) aged six to eleven months will be recruited. Data will be collected at baseline, and monthly thereafter for a period of six months. Participants will be provided with a monthly supply of the food to which their village has been allocated.

**Discussion:**

There is an urgent need to develop locally produced and culturally acceptable RUSFs, and to compare these with existing options in terms of their potential for preventing malnutrition, in Cambodia and elsewhere. This trial will contribute much-needed data on the effectiveness of supplementary foods with an animal-source food other than milk, by comparing a novel RUSF based on fish to one that uses milk (CSB++). Moreover, it will deepen the understanding of the impact of multiple micronutrients provided with or without macronutrients, by comparing the novel RUSF and CSB++, which combine macronutrients with multiple micronutrients, to Sprinkles, which contains no macronutrients. In addition, it will augment the body of evidence from Asia.

**Trial registration:**

ClinicalTrials.gov, Identifier: LNS-CAMB-INFANTS-EFF; NCT02257762.

## Background and rationale

Undernutrition is an underlying cause in almost half of all deaths in children under five years [1]. In Cambodia, rates of malnutrition remain stubbornly high [2] with 32% of all children under five years (and 40% of three to four year-olds) stunted, 10% wasted, and 24% underweight [2]. Suboptimal infant feeding practices, in particular, poor complementary feeding, result in inadequate energy and nutrient intakes to achieve optimal growth and micronutrient status, and consequently, childhood malnutrition.

Adequate complementary feeding can prevent malnutrition [3]. In Cambodia, the traditional weaning food is a white rice porridge called *borbor,* which has very low nutrient density. Special supplementary foods, containing a source of protein and lipids such as powered milk, soy or peanuts, and multiple micronutrients, can be used to improve complementary feeding. Supplementary foods can be either foods requiring preparation e.g. fortified blended products, such as Corn-Soy Blend++ (CSB++, now called Supercereal Plus), that is mixed with water to make a porridge; or ready-to-use e.g. biscuits, such as BP5. Until fairly recently, prevention of malnutrition relied on fortified blended products. However, increasingly, ready-to-use foods are lipid-based nutrient supplements (LNSs) which are often pastes, such as the peanut-based Plumpy’Doz™. These energy-dense ready-to-use supplementary foods (RUSFs) contain both macro and micronutrients [4, 5]. These new RUSFs are proving effective, as they have a higher energy content, and have a longer shelf life, and, since they require no preparation, are more convenient [5, 6]. Another common nutrition intervention is multiple micronutrient supplements such as Sprinkles, used solely to combat micronutrient deficiencies. These are individually-packed powders that can be added to food. Micronutrients are more likely to achieve growth outcomes when they are combined with energy, for example, in lipid-based nutrient supplements; there is no evidence that micronutrient powders alone contribute to sustained improvements in linear growth [7–12].

In Cambodia, various supplements and supplementary or therapeutic foods, including micronutrient powders, CSB++, BP100, and Plumpy’Nut™ have been used or trialled. The United Nations World Food Program (WFP) in Cambodia distributed CSB++ to children under two years to prevent malnutrition. Micronutrient powders (Sprinkles) have also been distributed through the public health system, though coverage is limited. These products are relatively expensive to procure and ship to Cambodia and in the case of Sprinkles, are not as effective as foods that contain macronutrients [9, 10]. Plumpy’Nut™, which is produced in France by Nutriset, was trialled in Cambodia in 2009 and was not well accepted [13]. Nor was CSB++ very well accepted in practice [14]. Due to lack of acceptability, and also due to budget constraints, WFP phased out distribution of CSB++ in Cambodia in June 2014. In addition, BP100, which is currently used in Cambodia to treat severe acute malnutrition, has not been well accepted [15].

For these reasons, the Cambodian Ministry of Health sought a ready-to-use food (in both therapeutic and supplementary versions) containing macro and micronutrients that was locally-produced and therefore more likely to be acceptable and cheaper than imported products.

In 2009 in Vietnam, UNICEF, IRD (Institute of Research for Development) and the National Institute of Nutrition developed a supplementary food from mainly local ingredients including rice, soy, mung beans, sugar, milk powder, oil and multiple micronutrients (called HEBI). This product proved more acceptable and as effective as Plumpy’Nut™ and is now widely used in Vietnam [16]. In mid-2013, IRD, partnering with UNICEF and the Cambodian Department of Fisheries Post-harvest Technologies and Quality (DFPTQ), began developing a novel RUSF based on the same concept as the Vietnamese product.

WHO recommends daily consumption of animal-source foods for their high protein, energy, and micronutrient availability, which plant-based complementary food alone cannot provide [17]. Animal-source foods have been associated with greater micronutrient status, linear growth and non-fat mass gain compared to non-animal-source food [18, 19]. Usually, milk or whey powder is the animal-source food most commonly used in supplementary foods including CSB++ and various RUSFs [20, 21]. However, milk powder is an expensive (and often imported) ingredient. Therefore, it could perhaps be replaced with a cheaper, local source of animal protein that is widely accepted by the target population. There are precedents for replacing milk in supplementary foods for cost effectiveness [22], but until now, only a handful of supplementary foods have used meat, fish or eggs and they have generally not been tested for effectiveness on a wide scale [23–27]. The only known effectiveness study involving a supplementary food with fish compared a peanut and soy-based fortified spread to a corn porridge fortified with fish powder with six to eighteen month old children, and found that children consuming the porridge with fish powder gained less weight from six to eleven months, but otherwise the two supplementary foods performed similarly in terms of weight gain from twelve to eighteen months and linear growth [28]. In a number of studies, supplementing with meat or milk (as opposed to a supplementary food product containing no meat or milk), the nutritional improvement was less than expected, and sometimes was only the slowing of growth faltering [29, 30]. A study in Kenya is the only one to have compared milk and meat, and found meat had a greater impact, perhaps because milk inhibited iron and zinc uptake [29]. In all of these studies, the limited impact of meat or milk may have been because the additional food did not contain a sufficient range or quantity of micronutrients to overcome deficiencies.

In Cambodia, fish is inexpensive, readily available and highly acceptable to local tastes, and could therefore replace milk in a supplementary food. Combined with rice, soy, mung beans, oil and sugar, the resulting product should be less expensive and more acceptable to Cambodians. IRD developed the first version of this product in paste form in early 2014. It was compared to BP100, and found to be equally acceptable in younger children, although older children preferred BP100’s milky taste to the fishy flavour of the RUSF.

The product was revised to reduce the fish smell and make it into a snack. It was then tested for acceptability in comparison to CSB++ and Sprinkles with *borbor* in June 2015. That trial demonstrated that children will eat the RUSF and that caregivers will feed it to their children. The next step is to assess the effectiveness of the food in preventing malnutrition, and promoting optimal growth and development. Therefore, a six-month effectiveness trial will be conducted. The impact of the product on children aged six to seventeen months will be compared to the impact of CSB++, Sprinkles, and to a control group consuming an unsupplemented diet, typically *borbor* at an early age (e.g. six to nine months) and thereafter, family foods.

## RUSF formulation

The RUSF has been formulated and produced locally, using local inputs. Small freshwater fish were cleaned, dried, roasted and ground. Soy and mung beans were roasted and ground, then mixed with the fish and coconut. This mix was extruded, then combined with multiple micronutrient premix (DMS), icing sugar, maltodextrin and canola oil to create the RUSF paste. Wafers were hand-made from rice flour, eggs, water, sugar, salt and coconut with small amounts of vanilla or sesame seeds for added flavour. The wafer is a hollow cylinder between 8.5-9 cm long with an internal diameter of 0.4–0.5 cm. Such wafers, unfilled, are a popular Cambodian snack. The ingredients of the RUSF are detailed in Table 1.

**Table 1:** Ingredients of RUSF snack (paste and wafer)

Ingredients	g/100 g
Small indigenous fish	5.9
Mung beans	9.6
Rice	4.2
Soy beans	12.2
Icing sugar	10.3
Maltrodextrin	9.3
Canola oil (g)	3.7
Palm vegetable shortening	14.0
Desiccated coconut	1.5
Rice bran	2.2
Vitamin and mineral mix	0.9
Rice flour	9.0
Duck eggs	2.5
Refined sugar	7.2
Coconut	7.2
Salt	0.0
Flavour (vanilla or sesame seeds	0.1
Oil for cooking	0.4

Although there are no definitive guidelines for supplementary foods, the RUSF was developed with the recommended guidelines for the nutritional composition of RUTFs in mind [31]. RUTFs should provide 520-550 kcal/100 g with 10–12% and 45–60% of the total energy coming from proteins and lipids respectively. Our RUSF contains 484 kcal/100 g, with 11% and 45% of the total energy coming from proteins and lipids respectively. The energy content of the paste is 499 kcal/100 g. The wafer, filled with the RUSF paste, yields a final snack weighing approximately 10-11 g, including 7 g of paste and 3-4 g of wafer, with an energy content of approximately 48 kcal/piece.

All processing has been conducted in quality-certified facilities. The novel RUSF will be tested for microbiological safety (first five batches and every fifth batch thereafter) at the Pasteur Institute in Phnom Penh.

## Design and methods

### Objective and Hypothesis

This trial aims to evaluate the effectiveness of the locally produced RUSF on children aged six to seventeen months in preventing growth faltering and improving micronutrient status. The impact of the product will be compared to CSB++, Sprinkles, and to a control group consuming an unsupplemented diet.

Based on trials with other RUSFs, and the Cambodian experience with CSB++ [14], it is expected that this novel RUSF will be as effective as CSB++, and more effective than Sprinkles or the standard diet in promoting growth and preventing stunting [32, 33].

### Trial Design

The trial is a prospective, cluster randomised, non-blinded controlled trial among infants six to seventeen months of age. The trial aims to establish the superiority of the novel RUSF, using CSB++, and Sprinkles as active comparators and the unimproved diet as a control. The allocation ratio is 1:1. The study will take place over six months.

### Comparators

The RUSF will be compared with:

1. CSB++: CSB++ has been chosen as a comparator because it is currently the standard supplementary food. WFP usually provides CSB++ for children aged six months to two years to prevent malnutrition.
2. Sprinkles: Sprinkles micronutrient powders have been chosen since they are a commonly provided supplement in developing countries, such as Cambodia, with low dietary diversity, and complementary foods with low nutrient density [34].
3. Control: An unsupplemented diet, typically *borbor* and family foods, has been chosen as a control because this is the standard diet in Cambodia. *Borbor* is the traditional food for weanlings (children transitioning from exclusively milk diets to diets that include complementary foods) and is often the only food given until about nine months.

The active comparators comply with WFP and UNICEF standards for supplementary foods, and have been used and tested in Cambodia and elsewhere [18, 33, 35]. They have been found to be safe and to have no unintended side-effects. The table below contrasts the characteristics of the RUSF and comparators (Table 2).

**Table 2:** Characteristics of the RUSF and comparators

CHARACTERISTIC	RUSF	CSB++	Sprinkles
Daily serving size	40-110g*	100 g dry CSB++	1 sachet (1 g)
Animal-source food	Fish	Milk	–
Energy (kcal/100 g)	484	410	–
Protein (g/100 g)	13	16	–
Carbohydrates (g/100 g)	52	67	–
Lipids (g/100 g)	24	9	–
Fibre (g/100 g)	1.6	3	–
Vitamin A	1080 μg	540 μg	400 μg
Vitamin D	60 μg	4.6 μg	5 μg
Vitamin B1 (thiamine)	0.59 mg	0.47 mg	0.5 mg
Vitamin B2 (riboflavin)	0.89 mg	0.84 mg	0.5 mg
Vitamin B6	0.84 mg	2.1 mg	0.5 mg
Phosphorus	474 mg	530 mg	–
Calcium	366 mg	260 mg	–
Pantothenic acid	1.75 mg	7.3 mg	–
Copper	1.6 mg	–	0.56 mg
Vitamin E	10.9 mg	9.8 mg	5 mg
Folic acid	230 μg	115 μg	150 μg
Iron	8 mg	8.9 mg	10 mg
Magnesium	137 mg		–
Vitamin B3 (niacin)	9.63 mg	7.2 mg	6 mg
Vitamin C	53.4 mg	100 mg	30 mg
Zinc	8.4 mg	7.5 mg	4.1 mg
Potassium	806 mg	990 mg	–
Vitamin B12	10 μg	2.3 μg	0.9 μg
Biotin	0.37 mg	–	–
Selenium	90 μg	–	17 μg
Iodine	–	60 mg	90 μg
Vitamin K	3 μg	115 μg	–
Taste	Fishy	Creamy, sweet, smooth [52]	Should not have a taste [53]
Preparation	No	10 mins cooking	No
Acceptability in Cambodia	Yes	Acceptable in trial [52], but not in practice [14]	Yes [35]
Effectiveness in reducing malnutrition	To be tested	Not inferior to peanut-based RUSFs, which are the most effective in promoting linear growth and weight gain [18, 33]	Improves micronutrient status but not linear growth or weight gain [5, 12, 35]
Intra-household sharing	To be tested	Yes [33]	None noted [35]
Packaging	To be determined	Packaging may encourage sharing [5, 39]	Looks like “medicine” thus may discourage sharing [5, 39]
Local production capacity	Unknown	None [5]	None
Cost	To be determined. Goal is <US$0.10/day	Less expensive than peanut-based RUSFs if produced locally [18], but also have to consider logistics, time to treat, relapse [39]	Very cheap to produce at US$0.025/daily dose [11], but also have to consider logistics

*RUSF daily serving size depends on the child’s age, i.e. 6-8 m – 4 pieces, 40 g; 9-11 m – 6 pieces, 60 g; 12-17 m – 11 pieces, 110 g

The potential comparators that will not be used are BP100 (because it is designed to treat severe acute malnutrition) and peanut-based RUSFs. The latter will not be included because they are thought to be less acceptable, and are too expensive from current producers. Moreover, including peanuts in a locally produced Cambodian RUSF is not advisable as local production standards may not be adequate to safeguard against aflatoxin contamination, given that the rate of aflatoxin contamination of peanuts in South-East Asia is probably quite high [36–38].

### Outcomes and their measurement

The main outcome of interest is anthropometric status, i.e. length/height-for-age (L/HAZ), weight-for-height (WHZ) and weight-for-age (WAZ), calculated through monthly weight and height measurements. A HAZ < − 2 indicates stunting, a WAZ < − 2 indicates underweight, and a WHZ < − 2 indicates wasting. A secondary outcome is children’s body composition. Body composition, like linear growth, gives an indication of the quality of nutritional recovery, inasmuch as non-fat or lean tissue growth requires balanced nutrition while fat gain requires only calories [39]. Body composition will be calculated using triceps and subscapular skinfolds [40, 41]. Another secondary outcome is biochemical status, including iron status and anaemia, infection measured by C-reactive protein (CRP) and alpha-2 acid glycoprotein (AGP), and parasite infestation. An additional outcome is cognitive development and achievement of developmental milestones.

### Blinding

This will be an open trial with no blinding, since the three foods will be visibly different to data collectors, caregivers and children. The principal investigator (who will do supervision in the field) and the staff administering the intervention will know which food has been allocated to a given village.

### Study setting

The study will be conducted in northern peri-urban Phnom Penh (Khan Russey Keo, Mekong Health District). This area has a large population of urban poor whose children experience higher than average rates of underweight and stunting [42, 43].

### Allocation sequence generation and concealment

Randomisation of the interventions will occur at site level. Using UNICEF data on health centre coverage, potential villages and their populations (including the expected population of children aged 6–11 months) will be listed. Villages receiving Sprinkles or CSB++ will be excluded based on information provided by UNICEF, WFP and the Ministry of Health. Small villages that are close to each other may merged into one site, or large villages split into multiple sites, in order to create sites of similar sizes. Sites will then be randomised to one of the arms.

Thus, participants will not be individually randomised. All subjects in a given site will be in the same intervention group to avoid potentially confounding social interaction, such as inter-household sharing of different foods, and to ensure better compliance [44]. Sites will be randomly allocated to one of the foods, using an Excel random number table and a randomised incomplete block design. The principal researcher will generate the allocation sequence. At least three sites will be allocated to each food.

### Sample size

The main outcome of interest is anthropometric status (WHZ, WAZ and L/HAZ), calculated through changes in weight and length/height for the novel RUSF in comparison to the CSB++, Sprinkles, and the control after six months of the interventions. An overall required sample size of 424 subjects, or 106 subjects per group, was calculated, based on the assumptions of a difference in mean z-scores of 0.1 between the groups (95%CI), an SD = 0.8, and an assumption that subjects provide five measurements (out of a possible total of seven), with a precision of 0.05, power of 0.8. This is in keeping with similar effectiveness studies which have aimed to detect a difference in mean z-scores of 0.16 between groups, assuming SD = 0.8 [45], or a difference in mean z-scores of 0.1 and SD = 0.8 [20]. Therefore, detection of difference in mean z-scores of 0.1 between groups is reasonable.

**Fig. 1:**
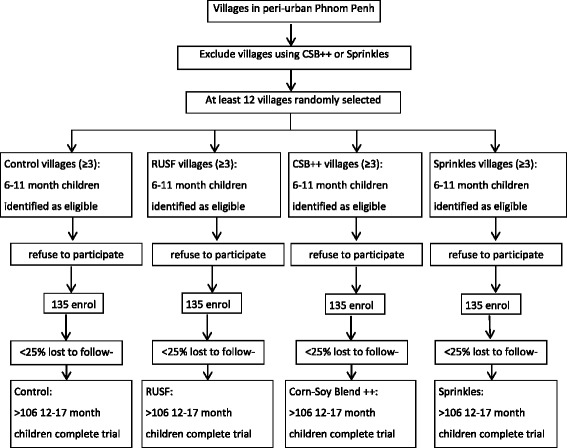
Site selection, recruitment and enrolment of children

In similar studies, retention has been high at [22, 28, 35]. Therefore, we can assume a maximum attrition of 25% with confidence. The total number of subjects enrolled will therefore be 530, or approximately 133 children in each group. This will be rounded up to 135 children/group for a total of 540 subjects, which should be adequate. See Fig. 1 for the planned site selection, recruitment and enrolment of children.

### Eligibility criteria

Children must be between six to eleven months of age at enrolment. It is expected that there will be approximately equal numbers of female and male children. Subjects must be normally nourished or only moderately malnourished (mid-upper arm circumference, MUAC> 115 mm, WHZ score > − 3), and healthy. Their iron status should be normal or only moderately anaemic; children with severe anaemia (haemoglobin, Hb < 70 g/l) will be referred for treatment. Any children who have been using Sprinkles or CSB++, are regularly consuming or receiving other food or micronutrient supplementation, are enrolled in any other research or supplementary feeding program, or have received therapy for acute malnutrition within one month prior to recruitment, will be excluded. Children with known food intolerances will also be excluded. Caregivers must be healthy, and must give informed signed consent for their children to be included.

### Recruitment, Enrolment and Consent

Village Health Support Group members (local health volunteers) will assist with recruitment, initially by inviting potential caregivers and children to participate in the trial. The data collection team will use a screening form to assess initial eligibility of participants. Some participants may be excluded at this point (e.g. on the basis of age or unwillingness to participate). Those who are excluded for severe acute malnutrition, anaemia or illness will be referred for treatment.

Those who are eligible will be provided with written and verbal information about the trial in Khmer language. If the participant is willing to continue, they will be asked to provide their verbal and signed (or fingerprinted) consent for them and their children to participate. It will be made clear that potential participants have the option of not joining the study. If they consent to participate, it will be made clear that they can ask questions, make complaints, or withdraw at any time.

### Data collection

After informed consent and enrolment, baseline data will be collected. This will include demographics, morbidity, anthropometry (weight, height, MUAC, skinfolds), biochemical samples (blood, stool), dietary data (breastfeeding, food frequency and dietary diversity), and developmental milestone achievement. Participants may still be excluded if they are malnourished (MUAC< 115 mm, WHZ score < − 3) or severely anaemic.

Participants in the intervention groups will then be provided with a one-month supply of the food to which their site has been allocated. Thereafter, participants will be provided with food on a monthly basis, and they will continue to consume the food over a six-month period. Data will be collected monthly (anthropometry, morbidity, developmental milestones), and/or at endline (biochemical).

Staff will inform the Village Health Support Group members in advance of monthly data collection sessions, and the latter will arrange for participants to be present. If caregivers are not present, they will be followed up by mobile phone and/or by the Village Health Support Group members and home visits will be conducted.

#### Anthropometric data

Anthropometric measurements will include weight to the nearest 0.1 kg, recumbent length to the nearest 0.1 cm, skinfolds to the nearest 1 mm, and MUAC to the nearest 1 mm. Weight will be measured with a SECA scale, length will be measured on wooden UNICEF height boards, MUAC will be measured with a flexible UNICEF insertion tape and skinfolds will be measured with a standard caliper (Holtain, United Kingdom). Anthropometry will be measured monthly. Children with MUAC < 115 mm and WHZ < − 3 at enrolment or at any time during the study will be excluded from the study and referred to the health centre for treatment.

#### Morbidity data

Data on diarrhoea and respiratory infections will be collected at the beginning of the study and monthly thereafter. Children with serious illnesses or severe malnutrition will be excluded from the study and referred to the health clinic for treatment.

#### Biochemical samples

Blood samples (4mls) will be drawn at baseline and endline by trained nurses who are skilled and experienced in taking paediatric blood samples. One to two drops will be used immediately to measure haemoglobin (using a HemoCue HB301 photometer). Of the remaining blood, 2mls will be placed in a trace element sodium heparin vacuette for further micronutrient analysis. The remaining blood will be placed in Ethylenediamine tetraacetic acid (EDTA) tubes for fatty acid analysis, then 40 μl will be pipetted onto pre-treated chromatography paper to be analysed as a dried blood spot. Blood samples will be stored, transported and analysed appropriately to avoid contamination and deterioration. Analysis will be conducted for micronutrient status including haemoglobin (g/l), ferritin (μg/l), transferrin receptor (mg/l), retinol-binding protein (vitamin A status) (μmol/l), zinc (μmol/l), C reactive protein (mg/l), using internationally accepted indicators [46].

Stool samples will be taken and tested for parasites. Stool containers will be distributed to caregivers and collected the following day. Analysis will be conducted using FLOTAX method.

#### Cognitive data and developmental milestones

The mental and motor development and behaviour of the participants will be tested monthly using the Bayley Scales of Infant Development (BSID), an internationally recognised standard of determining children’s developmental progress. In addition, a more detailed assessment will be conducted at endline.

#### Compliance data

Data on consumption, sharing, and adherence will be gathered monthly. Subjects will be provided with a month’s supply of food at this time.

#### Dietary data

Dietary data including breastfeeding status, food frequency and dietary diversity for caregivers and children will be collected monthly, using the Cambodian Demographic and Health Survey (CDHS) questionnaires as a model.

#### Endline data

Endline data will be collected on infants aged twelve to seventeen months at the end of the study.

### Timeline

The study will take place in 2016–2017 (see Table 3).

**Table 3:** Schedule of enrolment, interventions and assessments

	STUDY PERIOD					
	Enrolment	Allocation	Post-allocation			Close-out
TIMEPOINT	*Feb 2016*	*Feb 2016*	*Feb 2016*	*Mar-Sept 2016*	*Oct 2016*	*Early 2017*
ENROLMENT:	X					
Eligibility screen	X					
Informed consent	X					
Allocation		X				
INTERVENTIONS:						
*Baseline*			X			
*Monthly data collection*				X		
*Endline*					X	
DOCUMENTATION:						
*Anthropometry*						X
*Micronutrients*						X

### Statistical analysis

All data will be double-entered in Excel. Data will be analysed in the statistical software required by the PhD candidates’ respective universities. Thus, anthropometric data will be analysed by one PhD candidate in the statistical software STATA version 13.1, and biochemical and cognitive/developmental data will be analysed by another PhD candidate in SPSS and R.

Since most of the measures being taken are repeated on a monthly basis, the assumption of independence is not satisfied. Therefore, a mixed effect model, which is appropriate for repeated measurements, will be used. Predictor variables will be checked for normality and linearity, and manipulated and recoded as necessary. Outcome variables will be manipulated and recoded if necessary to deal with non-normality and/or for easier interpretation. Initial univariate screening will be conducted at *p* ≤ 0.2 level using simple logistic regression to screen for variables that could have an effect, and collinearity assumptions will be checked, in order to determine which covariates to include in the model. A complete mixed effects logistic regression will then be fit to the data. Significance levels will be considered *p* < 0.05. Any missing data will be treated as “missing at random” and accounted with the mixed effect model.

#### Anthropometric status

The main outcome of interest is change in anthropometric status. The independent variables are the food, sex and age, and the dependent variables are the mean weight-for-height (WHZ), height/length-for-age (H/LAZ) and weight-for-age (WAZ). Anthropometric indices for children will be calculated using World Health Organisation (WHO) 2006 standards (ANTHRO version 3.2.2 January 2011) and expressed as z-scores for weight-for-height (WHZ), height/length-for-age (H/LAZ) and weight-for-age (WAZ). Thus, multiple means will be compared, and changes will be analysed using a mixed effects model to determine whether there are statistically significant changes in WHZ, H/LAZ and WAZ of participants consuming the different foods.

#### Body composition

A second outcome is body composition measured by skinfold thickness. The independent variable is the food and the dependent variable will be the mean of skinfold thickness. The data will be analysed using a mixed effects model to determine whether there is a statistically significant difference in the body composition of participants eating the different foods.

#### Enrolment data

Enrolment data describing the characteristics of the recruited participants (e.g. sex, age, anthropometric and biochemical status, morbidity, breastfeeding status) will be reported as means ± SD for continuous measures.

## Ethics and consent

Ethics approval was received from the University of Queensland Medical Research Ethics Committee and the National Ethics Committee for Health Research (NECHR) in Cambodia. Written informed consent will be obtained from all the caregivers or parents of the participating children before recruitment into the study. Based on the experience of similar trials [20, 22, 28, 45], and given the inclusion and exclusion criteria, no harm is expected from trial participation. However, morbidity data will be collected every month, and this will record any harm (nausea, etc.) that could come from participation in the trial.

## Discussion

The development and comparison of new supplementary foods with current fortified blends and existing RUSFs in terms of their potential for preventing growth faltering and malnutrition responds to a need noted by various researchers [5, 22, 47–49] as well as to a programmatic need. Such products need to be affordable, effective, and acceptable in terms of preparation as well as taste [23]. The novel RUSF has proved acceptable and this trial will test its effectiveness, in terms of the main outcomes, namely, anthropometric measures, body composition and biochemical status.

Six months is sufficient time to see changes in the main outcome, that is, in anthropometric measures. Similar effectiveness studies (considering weight and length outcomes) have ranged from as little as four weeks [20], with many studies taking twelve weeks to compare three to eight food supplementation regimes [18, 22, 29, 32, 33, 50], and others taking six months [39]. The INCAP study in Guatemala provided supplementary food to children for up to seven years, but nevertheless noted a detectable difference after three and six months of supplementation [10]. With respect to linear growth, healthy infants grow approximately 1.25 cm each month from six to eleven months [51]. Golden notes that although the maximum rate of height gain is as yet unknown, catch-up growth can easily be three times the rate of normal growth. Thus, a malnourished child less than one year of age can gain one z-score in two to four weeks if receiving adequate nutrition [4]. The mean HAZ-score for a Cambodian child of six months is − 0.5, for twelve to seventeen month-olds is − 1.3 and for eighteen to twenty-three month-olds is − 1.8 [2]. Therefore, s/he loses around 0.8 z-score in six to eleven months, or approximately 0.07 z-score per month. In our trial, if the intervention stops or slows growth faltering, we could see a difference in HAZ-scores of up to 0.42 over six months between intervention and control groups.

Since it is not uncommon nowadays to find stunted but overweight individuals [4], a recent Cochrane review recommended that future research report results on body composition [49]. Among stunted, non-wasted children, prevention is preferable to treatment [5]. In either treatment or prevention of malnutrition, the aim is to increase non-fat mass (bones, muscles) in preference to fat mass, and one should therefore see linear growth as well as increased weight, since linear growth is a better indicator of nutritional recovery than weight gain [4]. Therefore, body composition will also be an important outcome of our trial.

The effectiveness trial of our novel RUSF will determine how a ready-to-use, fish-based, supplementary food compares with CSB++, Sprinkles, and an unsupplemented diet in terms of preventing of growth faltering and improving micronutrient status. This trial will contribute much-needed data on the effectiveness of supplementary foods with an animal-source food other than milk, by comparing a supplementary food with fish (the RUSF) and one with milk (CSB++). Moreover, it will deepen the understanding of the impact of multiple micronutrients provided with or without macronutrients, by comparing the RUSF and CSB++, which combine macronutrients with multiple micronutrients, and Sprinkles, which contains no macronutrients. Moreover, most studies on supplementary foods are from Africa, so this research will be an important contribution to the body of evidence from Asia [49].

There are two limitations of this trial. First, the data on consumption and compliance is based on self-reporting, and therefore risks response bias. It could be expected that any response bias would favour over-reporting of consumption, which may suggest lower effectiveness of the interventions. Similarly, sharing of foods with family members would likely be under-reported, again leading to underestimation of effectiveness. The second limitation is related to the generalisability of the findings to non-urban Cambodian populations, and to other South East Asian populations. Rural areas of Cambodia experience higher levels of malnutrition and poorer infant and young child feeding practices than urban [2]. Therefore, in rural settings, it would be difficult to predict if the interventions would appear more efficacious, or less.

From a programmatic point of view, if the novel RUSF proves successful, not only would it provide an acceptable, effective product for preventing childhood malnutrition. It might also simplify interventions in maternal and child nutrition in Cambodia and in countries where similar products could be produced, since, because of its composition, it could be used with pregnant and lactating women as well as children aged six months to two years.

## Abbreviations

AGP: Alpha-2 acid glycoprotein
BSID: Bayley Scales of Infant Development
CDHS: Cambodian Demographic and Health Survey
CRP: C-reactive protein
CSB++: Corn-Soy Blend Plus Plus, now called Supercereal Plus
DFPTQ: (Cambodian) Department of Fisheries Post-harvest Technologies and Quality
EDTA: Ethylenediamine tetraacetic acid
Hb: Haemoglobin
L/HAZ: Length/height-for-age z-score
LNS: Lipid-based nutrient supplement
MUAC: Mid-upper arm circumference
NECHR: National Ethics Committee for Health Research
RUSF: Ready- to-use supplementary food
RUTF: Ready- to-use therapeutic food
SD: Standard deviation
WAZ: Weight-for-age z-score
WFP: United Nations World Food Program
WHO: World Health Organisation
WHZ: Weight-for-height z-score
